# Immunotherapy or Targeted Therapy Versus Best Supportive Care for Advanced Gastric Cancer: A Systematic Review and Meta-analysis of Randomized Trials

**DOI:** 10.1007/s12029-024-01155-y

**Published:** 2025-03-04

**Authors:** Adriana Meade, Marilina Santero, Olga Savall-Esteve, Javier Bracchiglione, Leire Leache, Anna Selva, Ismael Macias, Paula Cerdà, Xavier Bonfill Cosp

**Affiliations:** 1 Iberoamerican Cochrane Centre, Institut Reserca Sant Pau (IR Sant Pau), Barcelona, Spain; 2 Autonomous University of Barcelona, Barcelona, Spain; 3 Interdisciplinary Centre for Health Studies (CIESAL), Universidad de Valparaíso, Viña del Mar, Chile; 4 Unit of Innovation and Organization, Navarre Health Service, Pamplona, Spain; 5 Navarre Institute of Health Research, Pamplona, Spain; 6 Institute of Research and Innovation Parc Tauli, Sabadell, Spain; 7 Corporació Sanitària Parc Taulí, Barcelona, Spain; 8 Hospital de la Santa Creu i Sant Pau, Barcelona, Spain; 9 CIBER Epidemiología y Salud Pública (CIBERESP), Barcelona, Spain

**Keywords:** Advanced gastric cancer, Immunotherapy, Targeted therapy, Systematic review, Meta-analysis, Non-chemotherapy anticancer drugs

## Abstract

**Purpose:**

To assess the efficacy and safety of non-chemotherapy anticancer drugs (immunotherapy or targeted therapy) compared to best supportive care (BSC) or placebo for the treatment of advanced gastric cancer (GC).

**Methods:**

Systematic review of randomized controlled trials (RCTs) searching (May 2022) MEDLINE, EMBASE, CENTRAL, Epistemonikos, ClinicalTrials.gov, and PROSPERO. Certainty of evidence was evaluated following GRADE.

**Results:**

Six RCTs included. Targeted therapies likely result in a slight increase in overall survival (OS) (HR 0.84, 95% CI 0.75, 0.93; moderate certainty) and progression-free survival (PFS) (HR 0.52, 95% CI 0.43, 0.62; moderate certainty). Toxicity had a slightly increased risk (RR 1.19, 95% CI 0.95, 1.48; low certainty). Immunotherapy also showed a likely improvement in PFS (HR 0.60, 95% CI 0.49, 0.73; moderate certainty), while toxicity showed a likely higher risk (RR 2.72, 95% CI 1.24, 5.94; moderate certainty). However, benefits in survival translated to time gains of slightly over a month for OS and less than a month for PFS. No data were reported on performance status (PS), hospital admissions, or quality of life (QoL).

**Conclusions:**

Our study suggests some survival benefits with low toxicity from these treatments, but gains are marginal. Uncertainties persist regarding their impact on QoL and outcomes for patients with poor PS. Caution is advised in treatment selection for advanced GC patients, who should actively participate in decision-making. Future research should include diverse patient populations and assess patient-centered outcomes with consistent comparator groups for BSC.

**Trial Registration:**

The study protocol was registered in OSF (https://doi.org/10.17605/OSF.IO/7CHX6) on 2022–04-01.

**Supplementary Information:**

The online version contains supplementary material available at 10.1007/s12029-024-01155-y.

## Introduction

Gastric cancer (GC) remains a significant cause of morbidity and mortality [1], ranking as the fifth most diagnosed and seventh most prevalent cancer worldwide [2, 3]. Over two-thirds of GC is diagnosed at an advanced stage. With an estimated survival of 5% [4], it is a leading cause of cancer death [5, 6].

The emergence of non-chemotherapy anticancer drugs, such as immunotherapy and molecular targeted therapies (IO + targeted), theoretically offers more potential benefits for advanced GC. These benefits might encompass controlling cancer-related symptoms, enhancing survival [4, 7–11], and personalized treatments. [12–14]. However, uncertainties persist regarding patient-centered outcomes beyond survival because achieving enduring disease control remains elusive [15, 16], with anticipated limited overall gains on a mid- or short-term basis [16]. Moreover, these therapies may entail toxicities and adverse events (AEs) that substantially impact patients’ quality of life (QoL) and functional status [17, 18]. Furthermore, the cost-effectiveness of these treatments poses challenges from both clinical and public health perspectives.

On the other hand, because of the uncertainty in the balance of disease control and therapy effects, often best supportive care (BSC) for advanced GC patients is used where there is a focus on symptom management without the use of anticancer drugs. BSC can provide a more comprehensive, early, personalized, and patient-centered approach, focusing on symptom management and improving the QoL [19], involving a less aggressive approach that can be particularly appropriate for advanced GC patients [19, 20].

Despite the increasing use of non-chemotherapy anticancer drugs, a comprehensive review comparing their efficacy and safety to BSC in advanced GC is currently lacking [21]. Thus, this review aims to fill this gap and provide valuable insights for optimal treatment decisions. The objective is to assess the efficacy and safety of non-chemotherapy anticancer drugs—IO + targeted—compared to BSC or placebo for the treatment of advanced gastric cancer.

## Methods

### Study Design

We conducted a systematic review (SR) in accordance with the Cochrane Handbook for Systematic Reviews and the Preferred Reporting Items for Systematic Reviews and Meta-Analyse (PRISMA) 2020 statement [22, 23] (Appendix 1). This SR represents the third stage of a larger project called the ASTAC-study, which aims to comprehensively synthesize evidence on the effects of systemic anticancer drugs compared to supportive care for individuals with advanced non-intestinal digestive cancers [21, 24–32]. The review protocol was prospectively registered in Open Science Framework (OSF) on April 1, 2022 [33].

### Eligibility Criteria

We included only randomized controlled trials (RCTs) that met all the following inclusion criteria: (1) included adult patients diagnosed with advanced or metastatic GC [34], considering studies that included patients with tumors located in various areas such as the cardia, fundus (corpus), body, antrum, pylorus, or gastroesophageal junction (GEJ), and encompassed any histological type, including adenocarcinoma and squamous cell carcinoma; (2) enrolled patients who received immunotherapy or molecular targeted therapy, either as monotherapy or in combination with other treatments, with or without supportive care; and (3) included a comparison group receiving any form of supportive treatment and/or placebo aimed at symptomatic or palliative control without any systemic anticancer treatment [19, 20].

We excluded studies focusing on neuroendocrine, stromal (GIST), or lymphatic neoplasms. Appendix 2 provides a summary of the eligibility criteria.

### Search Strategy

The search strategy derived from the broader ASTAC-study [33]. The following four databases were searched: MEDLINE (accessed through PubMed), EMBASE (accessed through OVID), the Cochrane Central Register of Controlled Trials (CENTRAL), and Epistemonikos. The initial search for the ASTAC study was conducted from the inception of each database up to December 2019. In addition, we searched Clinicaltrials.gov and PROSPERO for ongoing or unpublished data. An update of the search was performed in MEDLINE (PubMed) and CENTRAL up until May 2022. Furthermore, we manually reviewed the reference lists of relevant studies to identify any additional relevant articles [35]. Appendix 3 provides the search strategy.

### Selection and Data Extraction

Two reviewers performed an independent title and abstract screening. A third author resolved discrepancies when required. Afterward, two reviewers conducted the full-text screening, with a third author solving any disagreement. For all this process, we used the Rayyan platform [36].

Data extraction was carried out independently by two reviewers using a pre-designed and piloted structured template specifically created for this review. Any disagreements during extraction were resolved through discussion, involving a third author if necessary. The outcomes of interest in this SR were as follows: (a) Efficacy: overall survival (OS), progression-free survival (PFS); (b) Safety: toxicity and hospital admissions; and (c) Patient-centered outcomes: symptoms related to the disease, QoL, and quality of end-of-life care.

### Quality Assessment

Two reviewers independently assessed the risk of bias (RoB) of included studies using the Cochrane risk-of-bias tool for randomized trials [37]. The following six domains were assessed: 1) random sequence generation, 2) allocation concealment, 3) blinding of participants and personnel, 4) blinding of outcome assessment, 5) incomplete outcome data, and 6) selective reporting. The bias score was assessed as a judgment (high, low, or unclear) for individual elements from each domain, and we classified studies as low risk if they showed minimal risk of bias across all key domains, unclear if there were uncertainties in one or more domains, and high risk if one or more domains exhibited high risk of bias. Any disagreements were solved through consensus or, if necessary, with the involvement of a third reviewer.

### Statistical Analysis

For the analysis of time-to-event data, specifically OS and PFS, we utilized hazard ratios (HRs) with corresponding 95% confidence intervals (CIs). Regarding dichotomous data, such as OS and PFS at 6, 12, and 18 months and toxicity outcomes, we employed risk ratios (RRs) with 95% CIs. For the analysis of continuous data such as OS and PFS, we intended to use either the mean difference (MD) or the standardized mean difference (SMD) with 95% CIs. The MD compares the average difference in outcome values between two treatment groups. To ensure transparency and provide a comprehensive overview, we planned to report the absolute medians for individual studies when available. In cases where multiple studies were included, we presented the range of absolute medians to facilitate comparisons across studies. For the meta-analysis, we utilized a random-effects model. Subgroup analyses were conducted by the treatment group (molecular targeted therapy or immunotherapy). We performed all statistical analysis using Review Manager 5.4.1 software [38].

We expected heterogeneity in the studies due to diverse clinical and methodological factors, including participant characteristics (age, ethnicity, baseline ECOG performance status-PS, and treatment line). Differences in the type of drug interventions (molecular-targeted therapy, immunotherapy, intensity, or dose), outcome measures, and variability in the comparator and follow-up duration could also contribute to the observed heterogeneity. We used forest plots and the *I*^2^ index to assess this heterogeneity among the included studies in each analysis. A *I*^2^ value exceeding 75% was considered indicative of substantial heterogeneity.

### Certainty of the Evidence

We assessed the certainty of evidence of outcomes using the Grading of Recommendations Assessment, Development, and Evaluation (GRADE) approach [39]. The results of this assessment were presented in “summary of findings” (SoF) tables. Since our eligibility criteria considered only RCTs, we considered the initial certainty of evidence for each outcome as “high.” Then, we considered the following domains for potentially rating down this certainty: risk of bias, indirectness of the evidence, inconsistency among study results, imprecision of effect estimates, and potential publication bias. Finally, we classified the certainty of the evidence for each outcome as “high”, “moderate”, “low” or “very low” taking into account the downgrading factors mentioned above.

## Results

### Study Selection

**Fig. 1:**
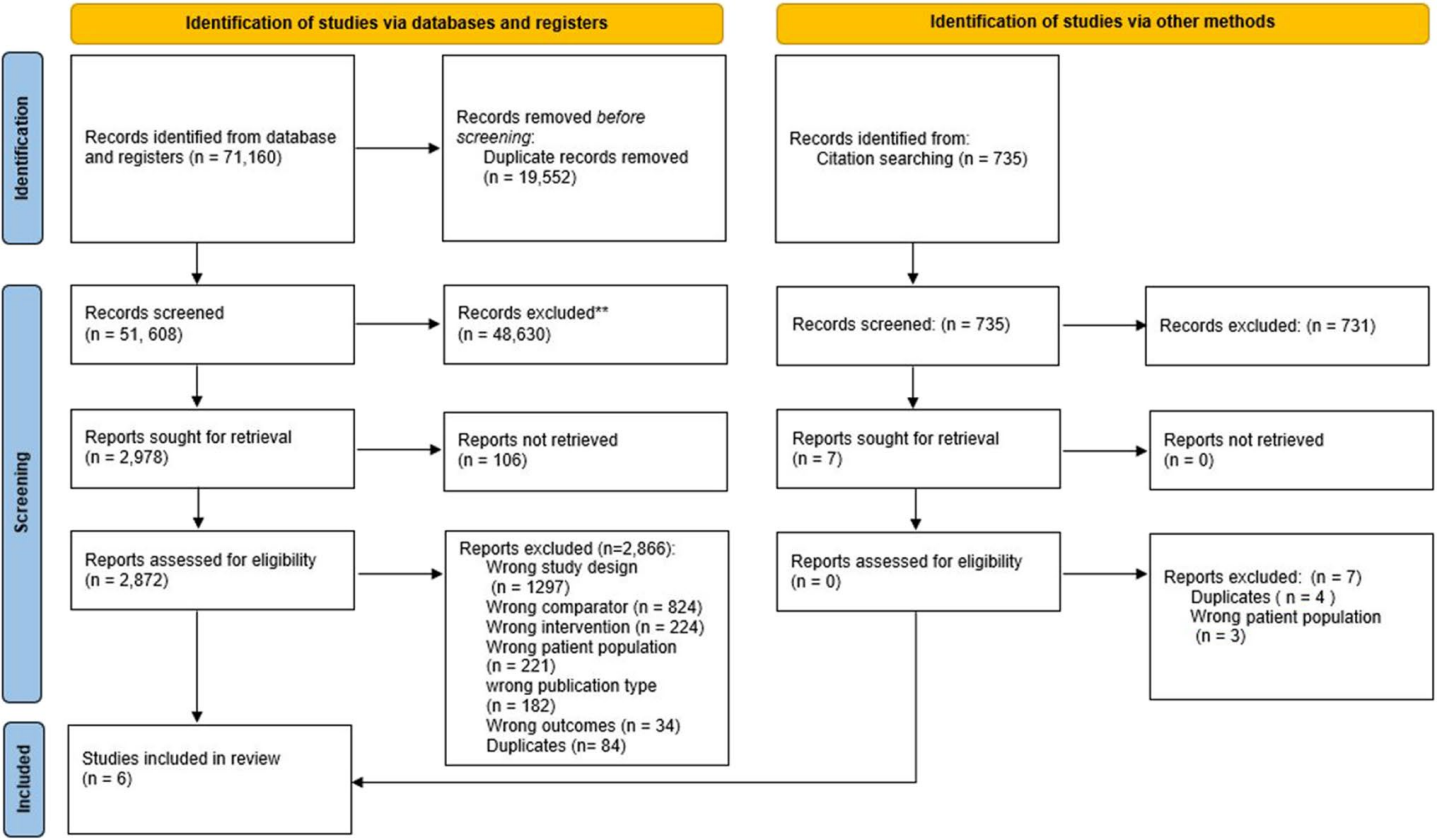
PRISMA 2020 flowchart

Our search identified 71,160 records for the initial stages of the comprehensive evidence synthesis project ASTAC-study [33]. After removing duplicates, we assessed 51,608 references by title and abstract, excluding 48,630 references. Therefore, we sought 2978 articles for full-text assessment, of which we included six RCTs [8, 12, 14, 40–42]. Figure 1 presents the PRISMA flow diagram, providing a detailed overview of the search results and the screening process.

#### Study Characteristics

Table 1 summarizes the general characteristics of the six included double-blind placebo-controlled RCTs. The selected studies spanned a period of 7 years, from 2013 to 2019, with five of them classified as phase III trials, and one as phase II trial [41]. Four trials were conducted in international settings involving multiple countries, while two focused specifically on East Asia [8, 14]. All studies were multicenter, with an average of 83 included centers and 2821 patients.

**Table 1:** Characteristics included studies (*n* = 6)

Study ID	Fuchs (2014)		Kang (2017)		Kang (2019)		Li (2016)		Ohtsu (2013)		Pavlakis (2016)	
Trial name/ registration	REGARD/NCT00917384		ONO-4538–12, ATTRACTION-2/NCT02267343		ANGEL/NCT03042611		NA/NCT01512745		GRANITE-1/NCT00879333		INTEGRATE/ACTRN12612000239864	
Study design	Double-blind placebo control		Double-blind placebo control		Double-blind placebo control		Double-blind placebo control		Double-blind		Double-blind placebo control	
Phase	III		III		III		III		III		II	
Arms	TT	Control	Immunotherapy	Control	TT	Control	TT	Control	TT	Control	TT	Control
Country (ies)	Multinational (29)		Japan, South Korea, and Taiwan		Multinational (13)		China		Multinational (22)		Multinational (4)	
Centers (*n*)	119		49		95		32		137		63	
*N* allocation	355		493		460		273		656		147	
	238	117	330	163	308	152	181	92	439	217	97	50
Age mean (range)	60 (52–67)	60 (51–71)	62 (54–69)	61 (53–68)	60.03	59.96	58 (23–71)	58 (28–70)	62 (20–86)	62 (26–88)	60 (32–85)	
Female %	29.0	32.0	29.4		23.3		25.0	24.2	27.0	26.0	20.0	20.0
Race/ethnicity	White 76%; Asian 16%; Black 2%; other 6%	White 78%; Asian 15%; Black 2%; other 6%	NR		Asian 67.8%; White 31.7%; Hispanic or Latino 1.1%; unknown or NR: 0.4%		NR		Asian 57%; White 38%; Black < 1%; other 4%	Asian 58%; White 35%; Black < 1%; other 7%	NR	
ECOG ≥ 2 (%)	1.0		0.0		0.0		0.0		0.1		0.0	
Follow-up (months)	12		18		36		30		18		6	
Line of therapy	2nd		3rd or more		3rd or more		3rd or more		2nd and 3rd		2nd or more	
Population	Adequate hematologic and organ function* Life expectancy of ≥ 12 weeks (GC, including GEJ)		Life expectancy of ≥ 12 weeks PD-L1 tumor expression was not required for patient enrolment (GC, including GEJ)		Ability to swallow oral medication Life expectancy of ≥ 12 weeks (GC, including GEJ)		Adequate hematologic and organ function (GC, including GEJ)		Adequate hematologic and organ function (GC, including GEJ)		Adequate hematologic and organ function Ability to swallow oral medication (GC, including GEJ)	
Intervention and comparator	Ramucirumab + BSC	Placebo + BSC	Nivolumab	Placebo	Rivoceranib + BSC	Placebo + BSC	Apatinib	Placebo	Everolimus + BSC	Placebo + BSC	Regorafenib + BSC	Placebo + BSC
	8 mg/kg IV every 2 weeks		3 mg/kg IV every 2 weeks. Three infusions one treatment cycle		700 mg VO QD. Each cycle duration 28 days		850 mg VO QD. Each cycle duration 28 days		10 mg VO QD		160 mg VO (four 40-mg tablets) QD. Each cycle duration 28 days	
Outcomes	OS, PFS, FS, toxicity, symptoms related to the disease, QoL		OS, PFS, toxicity,		OS, PFS, toxicity, QoL		OS, PFS, toxicity, QoL		OS, PFS, FS, toxicity, QoL		OS, PFS, toxicity, symptoms related to the disease, QoL	
Funding	Private (ImClone Systems)		Private (Ono Pharmaceutical and Bristol-Myers Squibb)		Private (LSK BioPharma)		Private (Merck, Amgen, Hai Wang, Jiangsu Hengrui Medicine, AstraZeneca)		Private (Novartis, Novartis, Sanofi-Aventis)		Public/Private (Bayer HealthCare Pharmaceuticals, Novartis, Genentech, Bayer Schering Pharma, Amgen, Celgene, Pfizer)	
Conflict of interest	Declared		Declared		Declared		Declared		Declared		Declared	

*GC* gastric cancer, *GEJ* gastroesophageal junction, *NR* not reported, *ECOG* Eastern Cooperative Oncology Group, *BSC* best supportive care, *IV* intravenous, *VO* oral administration, *QD* once daily, *OS* overall survival, *PFS* progression-free survival, *FS* symptom-related to the disease, *QoL* quality of life, *TT* targeted therapies

^*^Disease progression within 4 months of the last dose of first-line platinum-containing or fluoropyrimidine-containing chemotherapy for metastatic disease, or within 6 months of the last dose of platinum-containing or fluoropyrimidine-containing adjuvant treatment

In terms of participants’ characteristics, the reported median age ranged from 58 to 61 years, covering an age range from 20 to 88 years. Among studies that provided information on participants’ sex, a higher proportion of males was observed, ranging from 68.0 to 80.8%, consistent with known global incidence by sex of approximately 2:1 [43]. Nearly all patients in the trials were fit (ECOG 0–1), with only two studies enrolling a small percentage (1%) of patients with ECOG 2 [12, 40].

All trial participants were pre-treated with at least two lines of therapy. Four of the six studies allowed more than three lines. Notably, none of the studies selected participants based on specific biomarker expressions or programmed death-ligand 1 (PD-L1) scores.

Regarding the evaluated anticancer drugs, five of the RCTs studied molecular targeted therapies, including ramucirumab [12], rivoceranib [42], apatinib [14], everolimus [40], and regorafenib [41]. These targeted therapies were assessed in the context of second-line [12, 40, 41] and third-line [8, 14, 40, 42] therapies. Additionally, one double-blind placebo-controlled RCT tested a third-line immunotherapy regimen involving nivolumab [8]. It is worth mentioning that in the studies conducted by Kang and Li et al., the control group was treated only with placebo [8, 14]. However, in the remaining studies, the control group received a combination of placebo and BSC [12, 40–42].

Ultimately, five of the six studies [8, 12, 14, 40, 42] were fully funded by private entities, with the exception of one [41], which reported both public and private funding.

### Risk of Bias

Among the included studies, two were found to have an overall low RoB [12, 14]. Three studies were considered to have an overall unclear RoB [8, 40, 42], and one study was classified as having a high RoB primarily due to incomplete outcome data [41]. Appendix 4 details the RoB assessment.

### Outcomes

#### Overall Survival

**Fig. 2:**
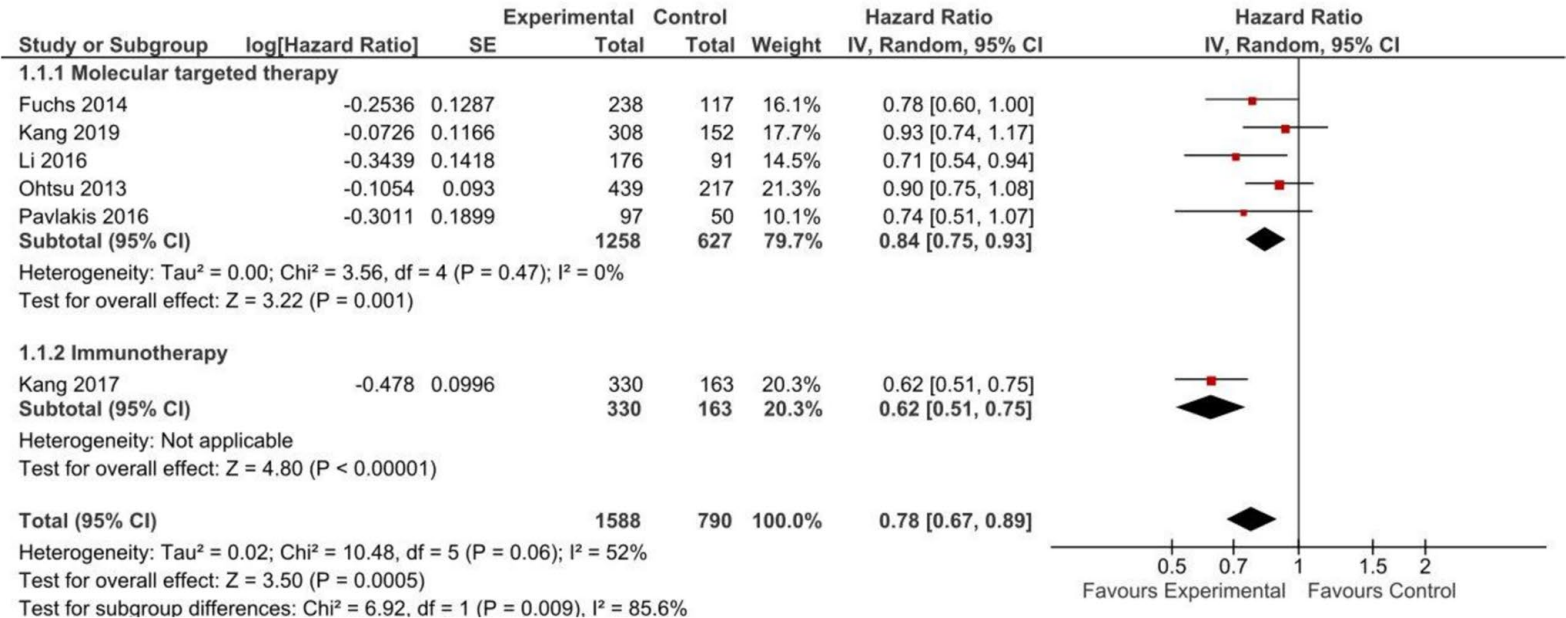
Overall survival in studies comparing non-chemotherapy anticancer drugs to supportive care or placebo for advanced gastric cancer by treatment type

The OS analysis included six studies [8, 12, 14, 33–35] (*n* = 2378). Non-chemotherapy anticancer drugs provide a statistically significant improvement in OS (HR 0.78; 95% CI 0.67, 0.89), both with molecular targeted therapy (HR 0.84, 95% CI 0.75, 0.93; moderate certainty) and immunotherapy (HR 0.62, 95% CI 0.51, 0.75; moderate certainty). Figure 2 presents the forest plot for the meta-analysis of OS. Appendix 8 provides the SoF table.

Our analysis of the absolute benefits likely increases the overall OS (MD 1.06; 96% CI 0.61, 1.51), with an MD of 1.04 months (95% CI 0.53, 1.55) for molecular targeted therapy [8, 12, 14, 41, 42] and an MD of 1.12 months (95% CI 0.15, 2.09) for immunotherapy [8]. Appendix 5 shows the meta-analysis for this outcome.

#### Progression-free Survival

**Fig. 3:**
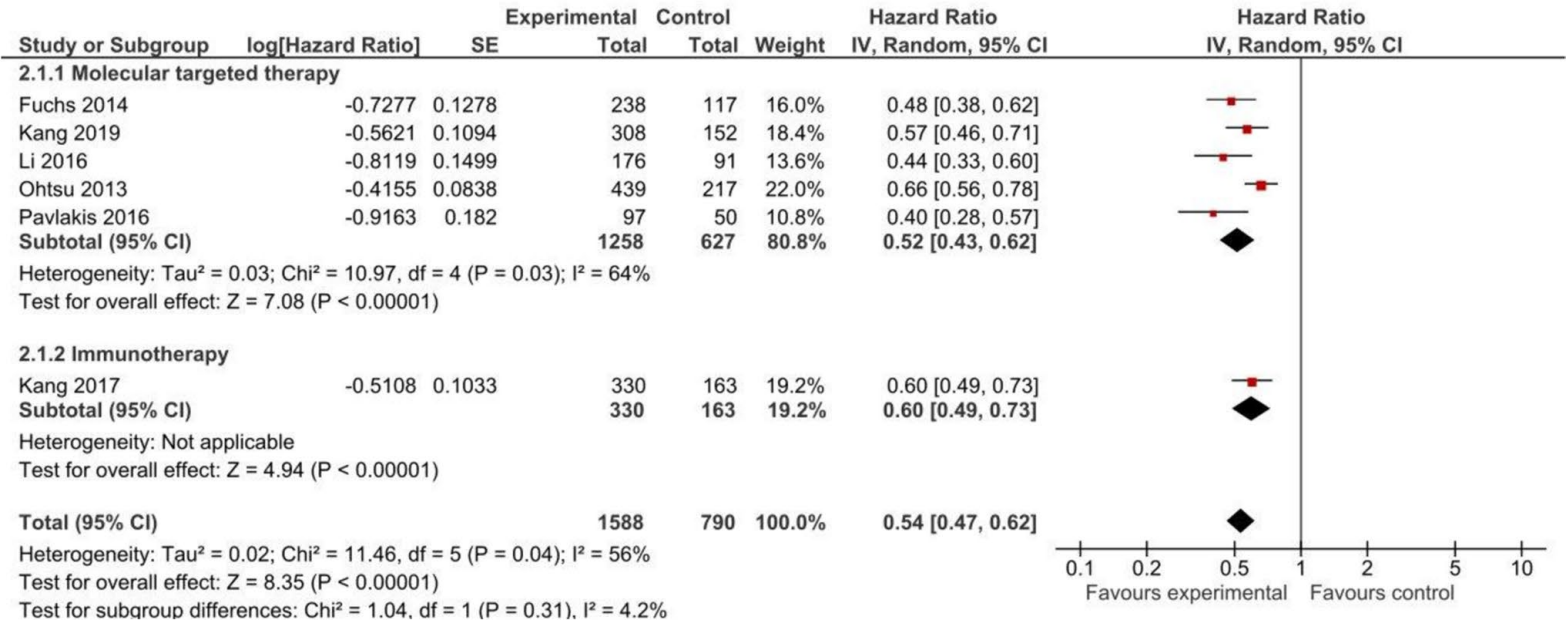
Progression-free survival in studies comparing non-chemotherapy anticancer drugs to supportive care or placebo for advanced gastric cancer by treatment type

PFS data from all the included RCTs [8, 12, 14, 40–42] were analyzed (*n* = 2378). Our meta-analysis of five RCTs involving 1885 patients for molecular targeted therapy likely shows an increase in PFS, with an HR of 0.52 (95% CI 0.43, 0.62; moderate certainty). Similarly, immunotherapy probably improves PFS with an HR of 0.60 (95% CI 0.49, 0.73; moderate certainty) based on data from one trial involving 493 patients. The results are visually presented in Fig. 3 and Appendix 8.

In absolute terms, molecular targeted therapy results in a slight increase in PFS (MD of 0.86 months 95% CI 0.63, 1.35), and immunotherapy probably results in little to no difference (MD of 0.16 months 95% CI 0.05, 0.27). PFS time probably favored the use of non-chemotherapy anticancer drugs, with a (MD of 0.65 months, 95% CI 0.30, 1.00; moderate certainty). Detailed results are shown in Appendix 6.

#### Toxicity

**Fig. 4:**
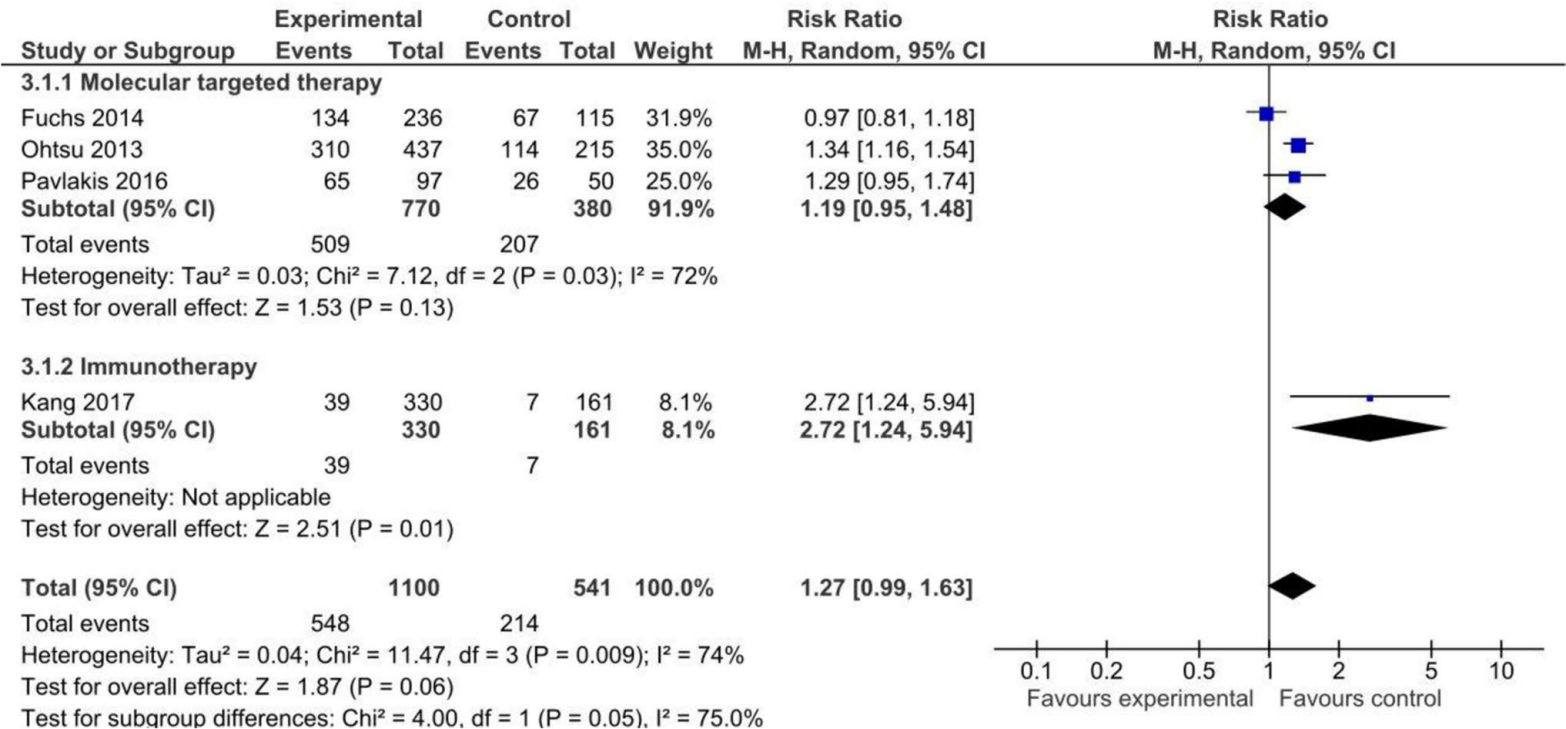
Toxicity in studies comparing non-chemotherapy anticancer drugs to supportive care or placebo for advanced gastric cancer by treatment type

Four [8, 12, 40, 41] of the included RTCs provided data on AEs of grade 3 or higher (*n* = 1641). Immunotherapy showed a likely higher risk of AEs than placebo with/without BSC, with only one [8] RCT with 46 events (RR 2.72, 95% CI 1.24, 5.94; moderate certainty). On the other hand, molecular targeted therapies pooled analysis of a non-statistically significant increase in the risk of AEs. The analysis based on three [12, 40, 41] trials with 762 events yielded no statistically significant differences (RR 1.19, 95% CI 0.95, 1.48; low certainty). Detailed results are shown in Fig. 4 and Appendix 8.

#### Quality of Life

The assessment of QoL using validated scales (EORTC QLQ-C30 (global and subscales), EORTC QLQ-STO22, EORTC QLQ-OG25, and EQ-5D-3L) was exclusively conducted in the RCTs focusing on molecular targeted therapies [12, 14, 40–42]. Due to heterogeneity in the reported outcomes among the included trials, we describe this outcome narratively. In Fuchs et al. [12] and Ohtsu et al. [40], the addition of molecular targeted therapy (ramucirumab and everolimus, respectively) to BSC compared to placebo plus BSC showed a trend towards a slightly longer time to deterioration in global QoL. However, these differences were not statistically significant. Neither Li et al. [14] nor Pavlakis et al. [41] demonstrated favorable results for QoL. Detailed results are shown in Appendix 7. None of the included studies provided data on hospital admissions, symptoms related to the disease (assessed separately from QoL), or quality of end-of-life care.

## Discussion

### Main Findings

This SR summarizes the results of six RCTs that assessed the efficacy and safety of non-chemotherapy anticancer drugs, including molecular targeted therapies (apatinib, everolimus, ramucirumab, regorafenib, and rivoceranib) and immunotherapy (nivolumab) with/without BSC compared with placebo with/without BSC in the treatment of advanced GC. The results likely suggest a small improvement in both OS and PFS in relative terms. Nonetheless, given the moderate certainty of evidence, it is crucial to approach these findings thoughtfully. Primarily, the benefits in OS in absolute terms likely result in mean time gains of only around one month (1.04 months for molecular targeted therapy and 1.12 months for immunotherapy), with less than a month of improvement in PFS. Nevertheless, it is worth remarking that a gain within a month or less represents a questionable clinical benefit.

Secondly, all the RCTs focused on advanced GC included only 1% of individuals within the ECOG PS ≥ 2 population. This cautionary approach advises against the inclusion of patients with higher ECOG grades, which, in turn, results in the absence of evidence for this population, which is expected to be the most predominant among advanced CG patients. The lack of data for patients with higher ECOG grades highlights a critical gap in understanding and addressing the needs of these individuals [44, 45]. This emphasizes the necessity for careful consideration of these therapies when offered to patients with poor PS in clinical practice due to the associated probable risks.

Furthermore, BSC was poorly defined in the control group and likely varied substantially across study sites in these large, mostly international trials. Overall, 66% included placebo plus BSC in their control group. This underscores the ongoing issue of lacking standardization in clinical trials, which may lead to overestimating the effect of comparator arms and call into question the validity of the data conclusions [19, 46, 47]. It also raises the possibility that standardizing BSC could further reduce the perceived effectiveness of active treatments. Despite ongoing discussions among scholars and healthcare decision-makers, there is still no universal consensus on defining and delivering BSC in RCTs [19].

Thirdly, despite an increased risk of AEs of grade 3 or higher with non-chemotherapy anticancer drugs (RR of 1.27), no difference in toxicity was found. However, the presence of moderate to substantial heterogeneity suggests that variability among the studies could impact confidence in the estimation and interpretation of results, highlighting the ongoing need to standardize the reporting of AEs. Additionally, we observed no effect of non-chemotherapy anticancer drugs on QoL. However, we were unable to combine study results due to heterogeneity in the measures used. Notably, none of the included studies reported data on other important patient-related outcomes such as hospital admissions, symptoms, or the quality of end-of-life care.

### Our Research in Context

In the context of research evaluating the effectiveness of emerging anticancer drugs, numerous studies, including those conducted by our ASTAC-study research group, have been undertaken [48–51]. Our ASTAC group’s investigations [21] have significantly contributed robust evidence, addressing previously unanswered questions and consistently producing reliable outcomes. However, it is important to note that none of these reviews have exclusively focused on evaluating the efficacy of immunotherapy and target therapy primarily in conjunction with supportive care measures. Moreover, this approach underscores patient-centered outcomes beyond mere survival or toxicity, which is particularly pertinent for individuals facing a high risk of short- or medium-term death, which excludes consideration of other anticancer treatments. Furthermore, our results claim for patient-centered outcomes beyond survival or toxicity, which holds particular importance for individuals facing a high risk of short- or medium-term mortality.

Our findings align with similar meta-analyses conducted by Chang et al. in 2017 and Rizzo et al. in 2020 [50, 51]. However, our study stands out by not only encompassing a meta-analysis of immunotherapy and target therapy but also evaluating the certainty of evidence based on the GRADE criteria [39]. Moreover, we incorporated a new study in the third-line treatment [42] that was not considered in their previous analyses. This additional study highlights the ongoing absence of clearly defined standard-of-care regimens in the third- or later-line setting for advanced GC, especially concerning the use of non-chemotherapy anticancer drugs.

Regarding AEs, our study uniquely focuses on AEs of grade 3 or higher severity. It is crucial to note that the distinct side effects associated with immunotherapy and molecular targeted therapy might contribute to the observed high heterogeneity in this aspect. Findings in the network meta-analyses by Cheng et al. in 2019 and Park et al. in 2021 [50, 51] suggest that immunotherapy may be a preferable and beneficial option, considering both efficacy and tolerability. Interestingly, these conclusions closely align with the outcomes of our research.

### Strengths and Limitations

This SR is helpful for clinicians and presents a robust assessment of the effect of non-chemotherapy anticancer drugs compared to BSC and/or placebo for patients with advanced GC, using a standardized approach. Notably, the review goes beyond the traditional focus on survival and toxicity outcomes, providing a more comprehensive evaluation of the interventions’ impact on patients’ well-being. A comprehensive search strategy was implemented to ensure inclusivity and minimize selection bias, without language or date restrictions. Additionally, two independent reviewers rigorously conducted the selection, data extraction, and analysis processes. The review adhered to GRADE guidelines [39] to assess the certainty of the evidence for each outcome, further enhancing the transparency and credibility of the findings.

Certain limitations should be acknowledged. Many new treatments in GC for relapsed or refractory disease seek accelerated approval through single-arm phase 2 trials and, therefore, could not be considered in this review. Also, the approval status varies across different markets. For example, ramucirumab has broader approval, whereas rivoceranib is limited to specific regions [52, 53].

It is intriguing to observe that many patients, despite receiving multiple lines of treatment in advanced stages, are often described as having good or perfect PS. Also, many patients will have already received immune checkpoint inhibitors in earlier lines of therapy, further clouding the risk/benefit calculation when considering nivolumab versus BSC, which adds to the uncertainty in clinical decision-making. The skewed representation of younger patients with good ECOG and the exclusion of those with specific comorbidities raise concerns about the generalizability of the findings to a broader target population. It is unclear if elderly patients or those with ECOG > 2 could benefit similarly from third-line treatment with non-chemotherapy anticancer drugs, posing questions about potential errors or bias. Moreover, despite the innovative therapies, there is a scarcity of reports on outcomes beyond survival [54]. Notably, the QoL lacks standardization, making it regrettable that these metrics were not consistently used when comparing novel therapies to BSC focused on improving cancer-related symptoms and QoL.

Furthermore, the substantial heterogeneity in included populations, considering factors such as geographic region, ethnicity, age, sex, and the line of treatment, demands cautious interpretation. Variations in histological patterns and molecular profiles have not been adequately considered in clinical trials [55–57], potentially introducing bias. Additionally, the absence of participant selection based on biomarker expressions limits tailoring treatments to specific subgroups, impacting response variations. In addition to the aforementioned limitations, we highlight the presence of industry-funded studies, which could raise the limitation with the possibility of financial bias [58–60].

## Conclusions

Our SR faces challenges in definitively establishing the efficacy and safety of immunotherapy and target therapy anticancer drugs compared to BSC in patients with advanced GC with very good PS. While these drugs likely result in a marginal to modest benefit in OS, PFS compared to BSC and/or placebo, evidence regarding QoL and toxicity remains inconclusive. It is important to interpret these results cautiously and encourage better shared decision-making. Considering the little existing effectiveness, even among the most selected advanced CG patients, it seems reasonable to be quite conservative when offering active treatment options to the majority of CG patients. We highlight the need to include wider populations and to use standardized scales to get better patient-centered outcomes reports with emerging therapies.

Further research, particularly improved RCTs involving diverse populations and better standardization of QoL metrics, symptom control definitions, AEs reporting, and cost evaluation, is essential for gaining a comprehensive understanding of the impact of non-chemotherapy anticancer drugs on patient QoL. This approach is crucial for informed decision-making and optimizing patient care. By considering a broad range of outcomes, decision-making among patients, researchers, and healthcare providers can be enhanced, allowing for personalized treatments tailored to meet the individual needs and values of patients with advanced GC [61].

## Supplementary Information

Below is the link to the electronic supplementary material.

Supplementary file1 (DOCX 1043 KB)

## Data Availability

The datasets generated during and analysed during the current study are available from the corresponding author on reasonable request.

## Abbreviations

AEs: Adverse events
ASTAC: Appropriateness of Systemic Oncological Treatments for Advanced Cancer
BSC: Best supportive care
CI: Confidence interval
ECOG: Eastern Cooperative Oncology Group
GC: Gastric cancer
GEJ: Gastroesophageal junction
GRADE: Grading of Recommendations, Assessment, Development, and Evaluations
HR: Hazard ratio
IO: Immunotherapy
OS: Overall survival
PFS: Progression-free survival
PS: Performance status
QoL: Quality of life
RCTs: Randomized clinical trials
RoB: Risk of bias
RR: Relative risk
SoF: Summary of findings
SR: Systematic review
