# Supplementary material for: Immunotherapy or Targeted Therapy Versus Best Supportive Care for Advanced Gastric Cancer: A Systematic Review and Meta-analysis of Randomized Trials
Source: J Gastrointest Cancer. 2025 Mar 4;56(1):75. doi: 10.1007/s12029-024-01155-y (PMC11876278; doi:10.1007/s12029-024-01155-y)
Supplement: Supplementary file 1 — Supplementary file1 (DOCX 1043 KB) [file 12029_2024_1155_MOESM1_ESM.docx]

# Appendices

**Appendix 1. PRISMA 2020 checklist**

| Topic | No. | Item | Location where item is reported |
| --- | --- | --- | --- |
| TITLE |  |  |  |
| Title | 1 | Identify the report as a systematic review. | title page |
| ABSTRACT |  |  |  |
| Abstract | 2 | See the PRISMA 2020 for Abstracts checklist |  |
| INTRODUCTION |  |  |  |
| Rationale | 3 | Describe the rationale for the review in the context of existing knowledge. | page 2 |
| Objectives | 4 | Provide an explicit statement of the objective(s) or question(s) the review addresses. | page 2 |
| METHODS |  |  |  |
| Eligibility criteria | 5 | Specify the inclusion and exclusion criteria for the review and how studies were grouped for the syntheses. | page 2 and appendix 2 |
| Information sources | 6 | Specify all databases, registers, websites, organisations, reference lists and other sources searched or consulted to identify studies. Specify the date when each source was last searched or consulted. | page 2 |
| Search strategy | 7 | Present the full search strategies for all databases, registers and websites, including any filters and limits used. | page 3 and appendix 3 |
| Selection process | 8 | Specify the methods used to decide whether a study met the inclusion criteria of the review, including how many reviewers screened each record and each report retrieved, whether they worked independently, and if applicable, details of automation tools used in the process. | page 3 |
| Data collection process | 9 | Specify the methods used to collect data from reports, including how many reviewers collected data from each report, whether they worked independently, any processes for obtaining or confirming data from study investigators, and if applicable, details of automation tools used in the process. | page 4 |
| Data items | 10a | List and define all outcomes for which data were sought. Specify whether all results that were compatible with each outcome domain in each study were sought (e.g. for all measures, time points, analyses), and if not, the methods used to decide which results to collect. | page 3-4 |
|  | 10b | List and define all other variables for which data were sought (e.g. participant and intervention characteristics, funding sources). Describe any assumptions made about any missing or unclear information. | page 3-4 |
| Study risk of bias assessment | 11 | Specify the methods used to assess risk of bias in the included studies, including details of the tool(s) used, how many reviewers assessed each study and whether they worked independently, and if applicable, details of automation tools used in the process. | page 3 |
| Effect measures | 12 | Specify for each outcome the effect measure(s) (e.g. risk ratio, mean difference) used in the synthesis or presentation of results. | page 3 |
| Synthesis methods | 13a | Describe the processes used to decide which studies were eligible for each synthesis (e.g. tabulating the study intervention characteristics and comparing against the planned groups for each synthesis (item 5)). | page 3-4 |
|  | 13b | Describe any methods required to prepare the data for presentation or synthesis, such as handling of missing summary statistics, or data conversions. | page 3-4 |
|  | 13c | Describe any methods used to tabulate or visually display results of individual studies and syntheses. | page 3-4 |
|  | 13d | Describe any methods used to synthesize results and provide a rationale for the choice(s). If meta-analysis was performed, describe the model(s), method(s) to identify the presence and extent of statistical heterogeneity, and software package(s) used. | page 3-4 |
|  | 13e | Describe any methods used to explore possible causes of heterogeneity among study results (e.g. subgroup analysis, meta-regression). | page 3-4 |
|  | 13f | Describe any sensitivity analyses conducted to assess robustness of the synthesized results. | page 3-4 |
| Reporting bias assessment | 14 | Describe any methods used to assess risk of bias due to missing results in a synthesis (arising from reporting biases). | page 3-4 |
| Certainty assessment | 15 | Describe any methods used to assess certainty (or confidence) in the body of evidence for an outcome. | page 4 |
| RESULTS |  |  |  |
| Study selection | 16a | Describe the results of the search and selection process, from the number of records identified in the search to the number of studies included in the review, ideally using a flow diagram. | page 5 and figure 1 |
|  | 16b | Cite studies that might appear to meet the inclusion criteria, but which were excluded, and explain why they were excluded. | NR |
| Study characteristics | 17 | Cite each included study and present its characteristics. | page 5 and table 1 |
| Risk of bias in studies | 18 | Present assessments of risk of bias for each included study. | page 9 and appendix 4 |
| Results of individual studies | 19 | For all outcomes, present, for each study: (a) summary statistics for each group (where appropriate) and (b) an effect estimate and its precision (e.g. confidence/credible interval), ideally using structured tables or plots. | page 9 to 11 and appendix 5, 6, 7, 8 |
| Results of syntheses | 20a | For each synthesis, briefly summarise the characteristics and risk of bias among contributing studies. | page 9 to 11 |
|  | 20b | Present results of all statistical syntheses conducted. If meta-analysis was done, present for each the summary estimate and its precision (e.g. confidence/credible interval) and measures of statistical heterogeneity. If comparing groups, describe the direction of the effect. | page 9 to 11 |
|  | 20c | Present results of all investigations of possible causes of heterogeneity among study results. | page 9 to 11 |
|  | 20d | Present results of all sensitivity analyses conducted to assess the robustness of the synthesized results. | page 9 to 11 |
| Reporting biases | 21 | Present assessments of risk of bias due to missing results (arising from reporting biases) for each synthesis assessed. | NA |
| Certainty of evidence | 22 | Present assessments of certainty (or confidence) in the body of evidence for each outcome assessed. | appendix 8 |
| DISCUSSION |  |  |  |
| Discussion | 23a | Provide a general interpretation of the results in the context of other evidence. | page 11 |
|  | 23b | Discuss any limitations of the evidence included in the review. | page 12 |
|  | 23c | Discuss any limitations of the review processes used. | page 12 |
|  | 23d | Discuss implications of the results for practice, policy, and future research. | page 13 |
| OTHER INFORMATION |  |  |  |
| Registration and protocol | 24a | Provide registration information for the review, including register name and registration number, or state that the review was not registered. | page 1, |
|  | 24b | Indicate where the review protocol can be accessed, or state that a protocol was not prepared. | page 1 |
|  | 24c | Describe and explain any amendments to information provided at registration or in the protocol. | page 2 |
| Support | 25 | Describe sources of financial or non-financial support for the review, and the role of the funders or sponsors in the review. | page 14 |
| Competing interests | 26 | Declare any competing interests of review authors. | page 14 |
| Availability of data, code and other materials | 27 | Report which of the following are publicly available and where they can be found: template data collection forms; data extracted from included studies; data used for all analyses; analytic code; any other materials used in the review. | page 14 |

##

## *From:* Page MJ, McKenzie JE, Bossuyt PM, Boutron I, Hoffmann TC, Mulrow CD, et al. The PRISMA 2020 statement: an updated guideline for reporting systematic reviews. MetaArXiv. 2020, September 14. DOI: 10.31222/osf.io/v7gm2. For more information, visit: www.prisma-statement.org

##

## **Appendix 2. Summarised eligibility criteria**

| **PICO** | **Inclusion criteria** | **Exclusion criteria** |
| --- | --- | --- |
| Patients | Adults (as defined by study authors) with an advanced primary NIDC, as defined by study authors or according to the following staging:  - GEJl junction: IIIb, IIIc, IV  - Gastric: IIIb, IIIc, IV | Neuroendocrine, stromal or lymphatic neoplasms. |
| Interventions | Any biological/targeted therapy, or immunotherapy either monotherapy or in combination with chemotherapy or another type of systemic oncological treatment, either individually or in combination.  The study also includes participants who may or may not have received supportive care along with these treatments. | Surgery or radiotherapy as sole interventions.  Adjuvant or neoadjuvant chemotherapy. |
| Comparisons | Any supportive treatment administered with the purpose of symptomatic or palliative control, including BSC, placebo, or non-specified control intervention. | Non-palliative treatments |
| Outcomes | Primary outcomes:   - Survival - Quality of life - Functional status - Toxicity   Secondary outcomes:   - Progression-free survival - Symptoms related to the disease - Admissions to hospital or long-term centre, or emergency consultations - Quality of end-of-life care |  |
| Type of studies | Randomised clinical trials | Quasi-experimental studies, observational studies, reviews, protocols. |

#

# **Appendix 3. Search strategy**

### Search strategy for MEDLINE/Pubmed

This appendix provides the search strategy for MEDLINE/Pubmed. The same search strategy structure was replicated in all databases.

For this systematic review, we performed two complementary search strategies:

- Search strategy A: Common for the whole ASTAC project. It includes terms broader than gastric cancer since it aims to inform overviews and mapping reviews of advanced, non-intestinal digestive cancers, in addition to this systematic review.
- Search strategy B: Update of search strategy A, focused on terms specific to this review.

### Search strategy A: MEDLINE/Pubmed for the overall ASTAC project (up to December 2019)

| #1 | ("Gastrointestinal Neoplasms"[Mesh:NoExp] OR "Esophageal Neoplasms"[Mesh] OR "Stomach Neoplasms"[Mesh] OR "Liver Neoplasms"[Mesh] OR "Biliary Tract Neoplasms"[Mesh] OR "Pancreatic Neoplasms"[Mesh]) |
| --- | --- |
| #2 | ((esophag*[Title] OR oesophag*[Title] OR stomach*[Title] OR gastric*[Title] OR gastroesophag*[Title] OR liver*[Title] OR hepatic*[Title] OR hepatocel*[Title] OR biliary tract*[Title] OR bile duct*[Title] OR gallbladder*[Title] OR gall bladder*[Title] OR pancreas*[Title] OR pancreatic*[Title] OR gastrointestinal*[Title]) AND (cancer*[Title] OR carcinom*[Title] OR neoplasm*[Title] OR tumor*[Title] OR tumour*[Title] OR malignan*[Title] OR adenocar*[Title] OR oncolog*[Title])) |
| #3 | (#1 OR #2) |
| #4 | ("Palliative Care"[Mesh] OR "Terminal Care"[Mesh] OR "Neoplasm Metastasis"[Mesh]) |
| #5 | (palliative*[Title/Abstract] OR end of life*[Title/Abstract] OR end of live*[Title/Abstract] OR terminal*[Title/Abstract] OR metasta*[Title/Abstract] OR BSC[Title/Abstract] OR supportive care*[Title/Abstract] OR advanced*[Title/Abstract] OR unresect*[Title/Abstract] OR irresect*[Title/Abstract] OR nonresect*[Title/Abstract] OR non resect*[Title/Abstract] OR inopera*[Title/Abstract] OR unopera*[Title/Abstract] OR nonopera*[Title/Abstract] OR non opera*[Title/Abstract] OR non-opera*[Title/Abstract] OR stage IV[Title/Abstract]) |
| #6 | (#4 OR #5) |
| #7 | (#3 AND #6) |
| #8 | ("Antineoplastic Protocols"[Mesh] OR "Chemoradiotherapy"[Mesh] OR "Induction Chemotherapy"[Mesh] OR "Maintenance Chemotherapy"[Mesh] OR "Consolidation Chemotherapy"[Mesh]) |
| #9 | (antineoplastic*[Title] OR antineoplasic*[Title] OR chemotherap*[Title] OR chemoradiotherap*[Title] OR radiochemotherap*[Title] OR carboplatin*[Title] OR cisplatin*[Title] OR oxaliplatin*[Title] OR platin*[Title] OR fluorouracil*[Title] OR 5-FU[Title] OR capecitabine*[Title] OR docetaxel*[Title] OR taxotere[Title] OR epirucibin*[Title] OR irinotecan*[Title] OR onivyde[Title] OR paclitaxel*[Title] OR abraxane[Title] OR trifluridine*[Title] OR tipiracil*[Title] OR lonsurf[Title] OR gemcitabine*[Title] OR gemzar[Title] OR mitomycin*[Title]) |
| #10 | (#8 OR #9) |
| #11 | ("Molecular Targeted Therapy"[Mesh] OR "Antibodies, Monoclonal"[Mesh] OR "Cancer Vaccines"[Mesh]) |
| #12 | (Target*[Title] OR antibod*[Title] OR immunotherap*[Title] OR vaccine[Title] OR vaccines[Title] OR vaccination[Title] OR tyrosine kinase inhibit*[Title] OR trastuzumab[Title] OR herceptin[Title] OR bevacizumab[Title] OR rilotumumab[Title] OR onartuzumab[Title] OR ramucirumab[Title] OR cyramza[Title] OR cetuximab[Title] OR panitumumab[Title] OR nimotuzumab[Title] OR claudiximab[Title] OR apatinib[Title] OR lapatinib[Title] OR regorafenib[Title] OR stivarga[Title] OR everolimus[Title] OR nivolumab[Title] OR opdivo[Title] OR pembrolizumab[Title] OR keytruda[Title] OR avelumab[Title] OR durvalumab[Title] OR ipilimumab[Title] OR checkpoint inhibit*[Title] OR cabozantinib[Title] OR cabometyx[Title] OR lenvatinib[Title] OR lenvima[Title] OR sorafenib[Title] OR nexavar[Title] OR sunitinib[Title] OR sutent[Title] OR erlotinib[Title] OR tarceva[Title] OR doxorubicin[Title]) |
| #13 | (#11 OR #12) |
| #14 | (#10 OR #13) |
| #15 | (#7 AND #14) |
| #16 | (animals [mh] NOT humans [mh]) |
| #17 | (#15 NOT #16) |

### Search strategy B: MEDLINE/Pubmed specific strategy update for this systematic review (from December 2019 until May 2022)

| #1 | ("Esophageal Neoplasms"[Mesh] OR "Stomach Neoplasms"[Mesh] ) |
| --- | --- |
| #2 | ((esophag*[Title] OR oesophag*[Title] OR stomach*[Title] OR gastric*[Title] OR gastroesophag*[Title] OR gastrointestinal*[Title]) AND (cancer*[Title] OR carcinom*[Title] OR neoplasm*[Title] OR tumor*[Title] OR tumour*[Title] OR malignan*[Title] OR adenocar*[Title] OR oncolog*[Title])) |
| #3 | (#1 OR #2) |
| #4 | ("Palliative Care"[Mesh] OR "Terminal Care"[Mesh] OR "Neoplasm Metastasis"[Mesh]) |
| #5 | (palliative*[Title/Abstract] OR end of life*[Title/Abstract] OR end of live*[Title/Abstract] OR terminal*[Title/Abstract] OR metasta*[Title/Abstract] OR BSC[Title/Abstract] OR supportive care*[Title/Abstract] OR advanced*[Title/Abstract] OR unresect*[Title/Abstract] OR irresect*[Title/Abstract] OR nonresect*[Title/Abstract] OR non resect*[Title/Abstract] OR inopera*[Title/Abstract] OR unopera*[Title/Abstract] OR nonopera*[Title/Abstract] OR non opera*[Title/Abstract] OR non-opera*[Title/Abstract] OR stage IV[Title/Abstract]) |
| #6 | (#4 OR #5) |
| #7 | (#3 AND #6) |
| #8 | ("Antineoplastic Protocols"[Mesh] OR "Chemoradiotherapy"[Mesh] OR "Induction Chemotherapy"[Mesh] OR "Maintenance Chemotherapy"[Mesh] OR "Consolidation Chemotherapy"[Mesh]) |
| #9 | (antineoplastic*[Title] OR antineoplasic*[Title] OR chemotherap*[Title] OR chemoradiotherap*[Title] OR radiochemotherap*[Title] OR carboplatin*[Title] OR cisplatin*[Title] OR oxaliplatin*[Title] OR platin*[Title] OR fluorouracil*[Title] OR 5-FU[Title] OR capecitabine*[Title] OR docetaxel*[Title] OR taxotere[Title] OR epirubicin*[Title] OR irinotecan*[Title] OR onivyde[Title] OR paclitaxel*[Title] OR abraxane[Title] OR trifluridine*[Title] OR tipiracil*[Title] OR lonsurf[Title] OR gemcitabine*[Title] OR gemzar[Title] OR mitomycin*[Title]) |
| #10 | (#8 OR #9) |
| #11 | ("Molecular Targeted Therapy"[Mesh] OR "Antibodies, Monoclonal"[Mesh] OR "Cancer Vaccines"[Mesh]) |
| #12 | (Target*[Title] OR antibod*[Title] OR immunotherap*[Title] OR vaccine[Title] OR vaccines[Title] OR vaccination[Title] OR tyrosine kinase inhibit*[Title] OR trastuzumab[Title] OR herceptin[Title] OR bevacizumab[Title] OR rilotumumab[Title] OR onartuzumab[Title] OR ramucirumab[Title] OR cyramza[Title] OR cetuximab[Title] OR panitumumab[Title] OR nimotuzumab[Title] OR apatinib[Title] OR lapatinib[Title] OR regorafenib[Title] OR stivarga[Title] OR everolimus[Title] OR nivolumab[Title] OR opdivo[Title] OR pembrolizumab[Title] OR keytruda[Title] OR avelumab[Title] OR durvalumab[Title] OR ipilimumab[Title] OR checkpoint inhibit*[Title] OR cabozantinib[Title] OR cabometyx[Title] OR lenvatinib[Title] OR lenvima[Title] OR sorafenib[Title] OR nexavar[Title] OR sunitinib[Title] OR sutent[Title] OR erlotinib[Title] OR tarceva[Title] OR doxorubicin[Title]) |
| #13 | (#11 OR #12) |
| #14 | (#10 OR #13) |
| #15 | (#7 AND #14) |
| #16 | (animals [mh] NOT humans [mh]) |
| #17 | (#15 NOT #16) |
| #18 | randomized controlled trial[pt] OR controlled clinical trial[pt] OR randomized[tiab] OR placebo[tiab] OR clinical trials as topic[mesh:noexp] OR randomly[tiab] OR trial [ti] |
| #19 | (#17 AND #18) |
| #20 | 2019/12/01:2022/05/06[pdat] |
| #21 | (#19 AND #20) |

# **Appendix 4. Risk of bias assessment**


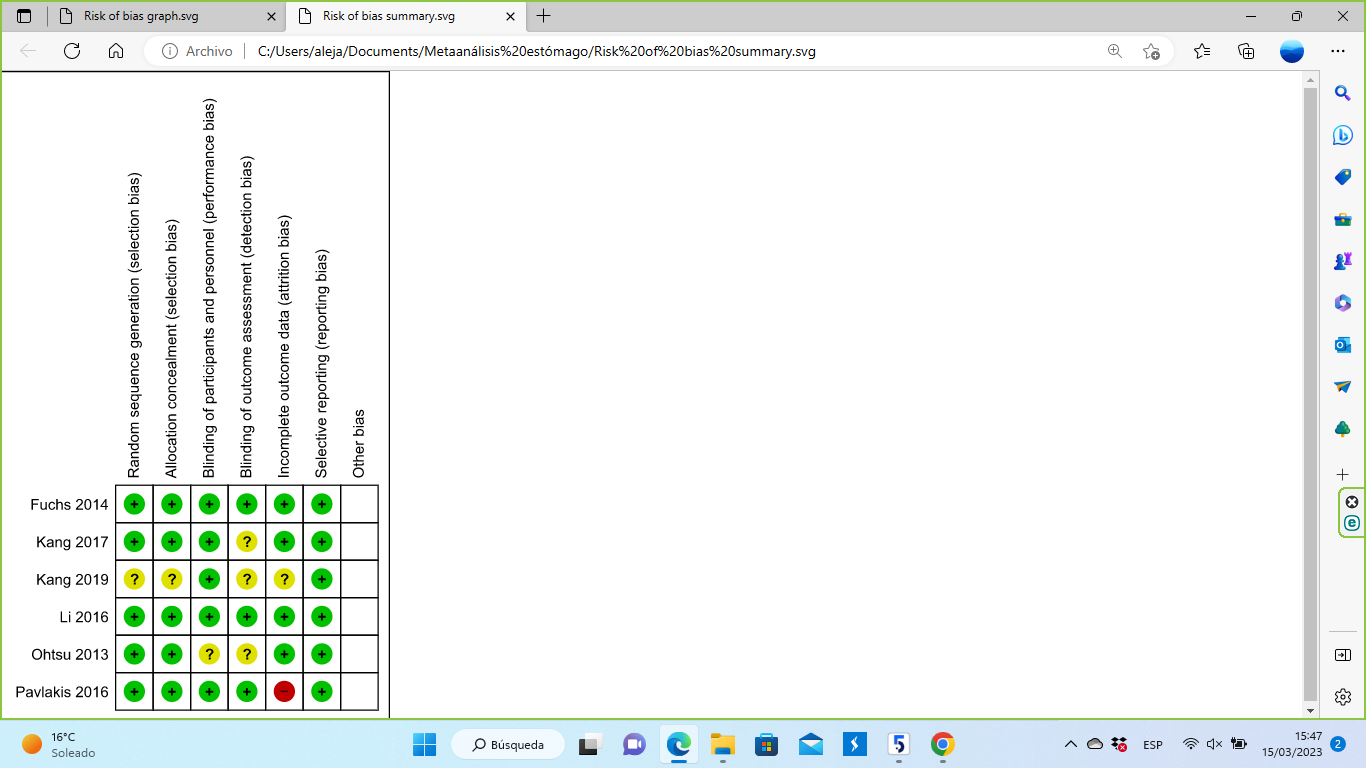


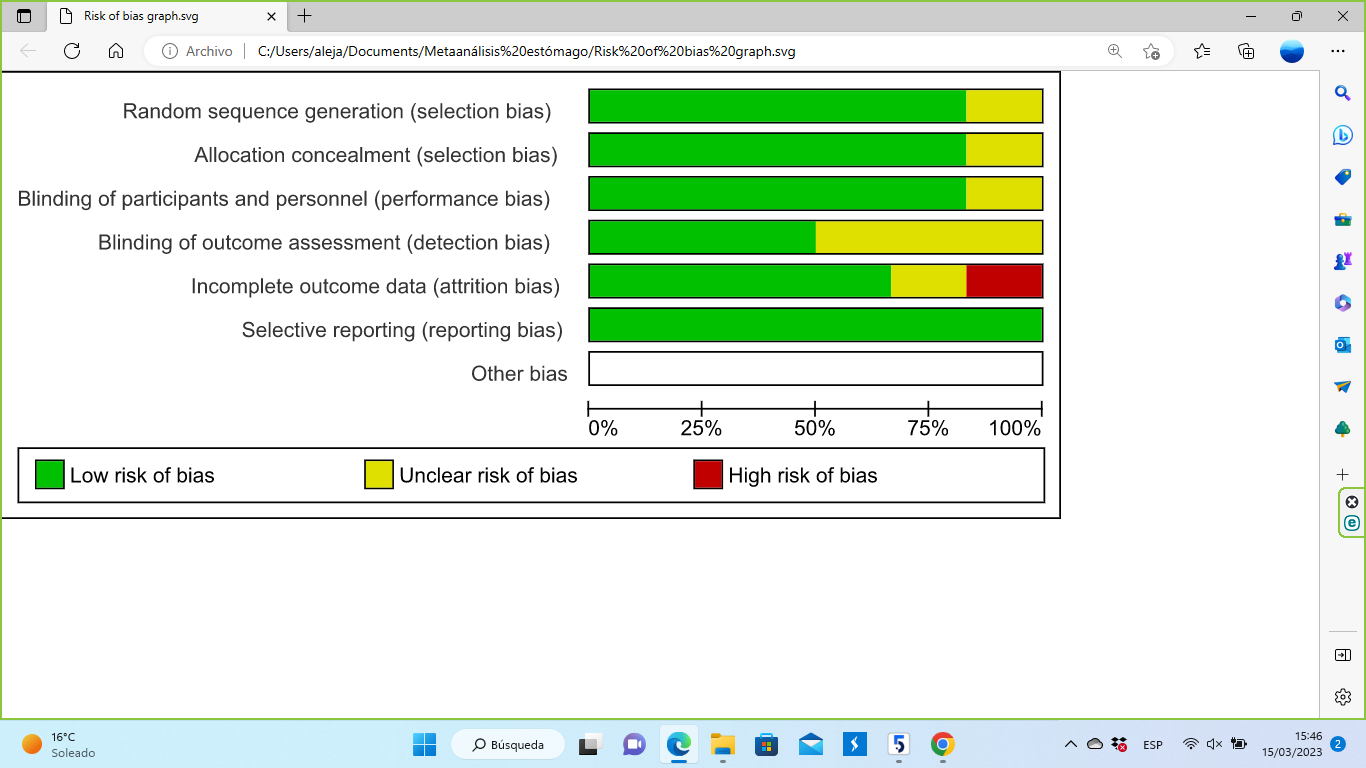


# **Appendix 5. Overall survival (OS) outcome: meta-analyses (continuous, 6, 12 and 18 months)**

OS as a continuous outcome


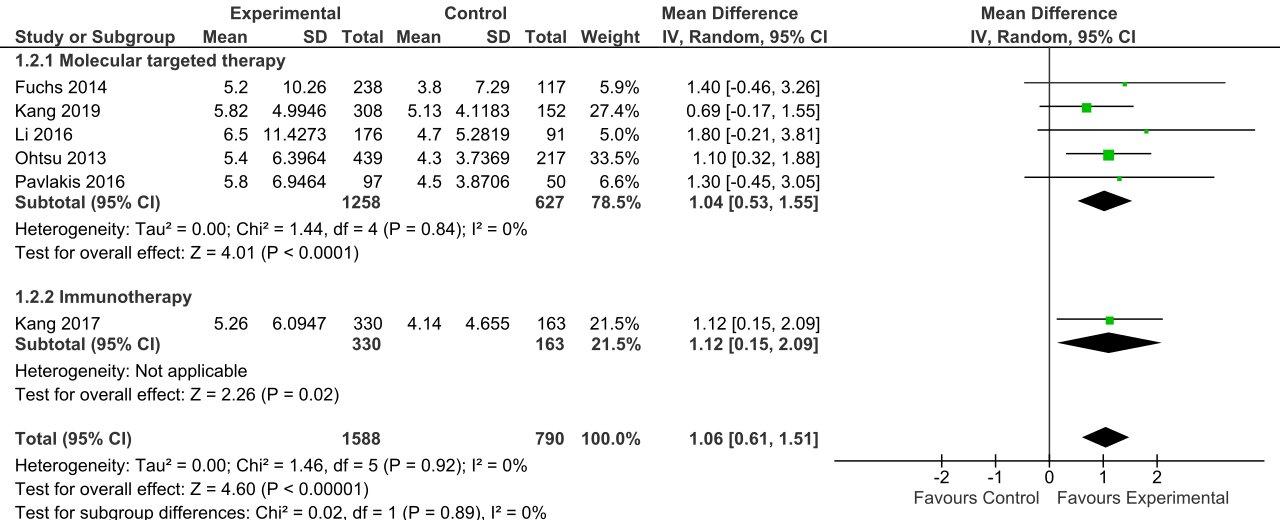


OS follow-up 6 months


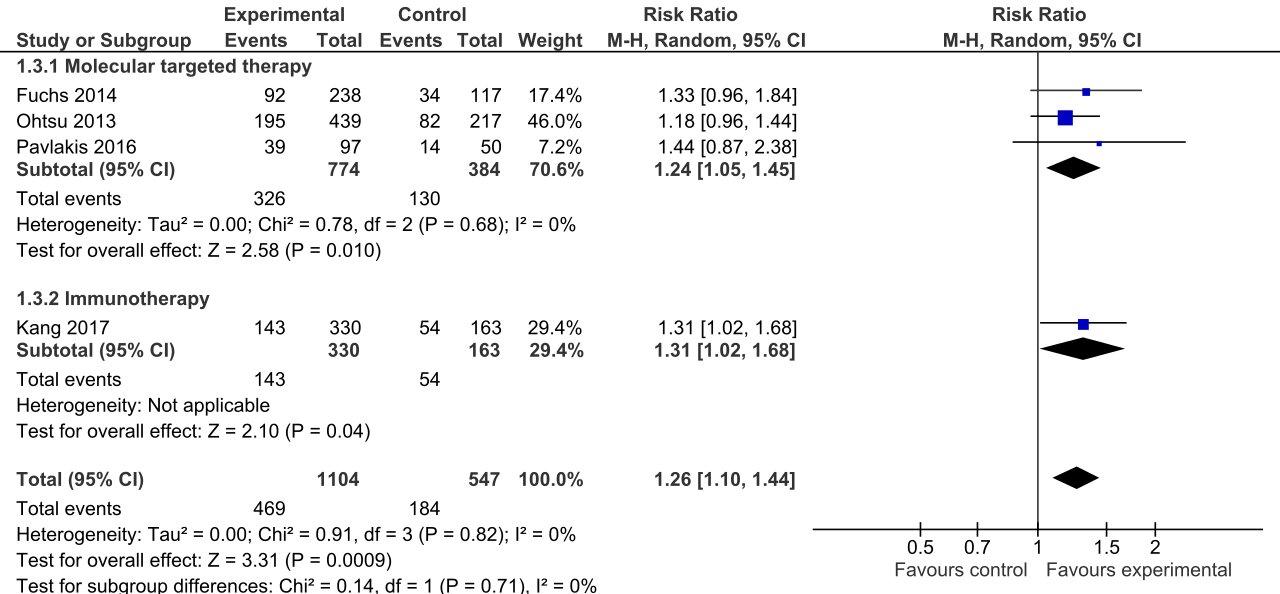


OS follow-up 12 months


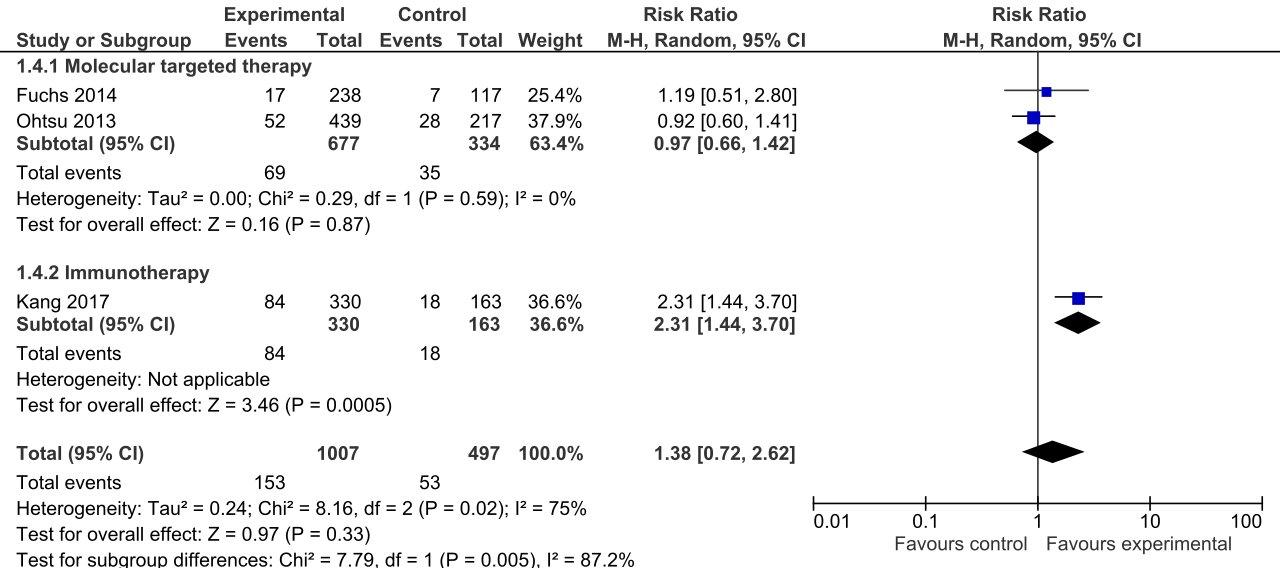


OS follow-up 18 months


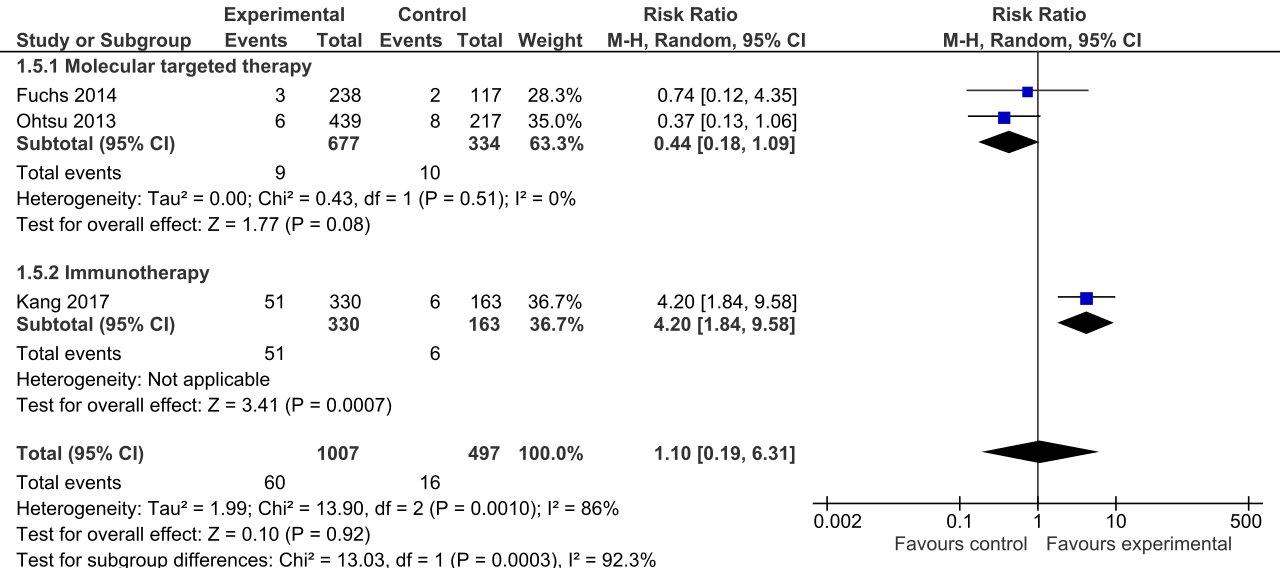


#

# **Appendix 6. Progression free survival (PFS) outcome: meta-analyses (continuous, 6, 12 and 18 months)**

PFS as a continuous outcome


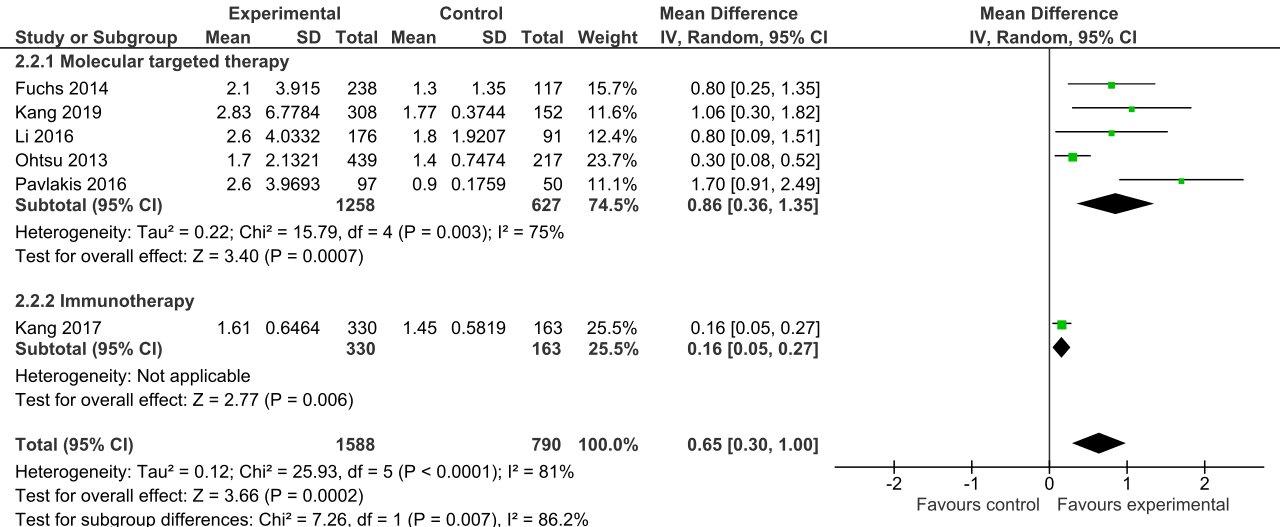


PFS follow-up 6 months


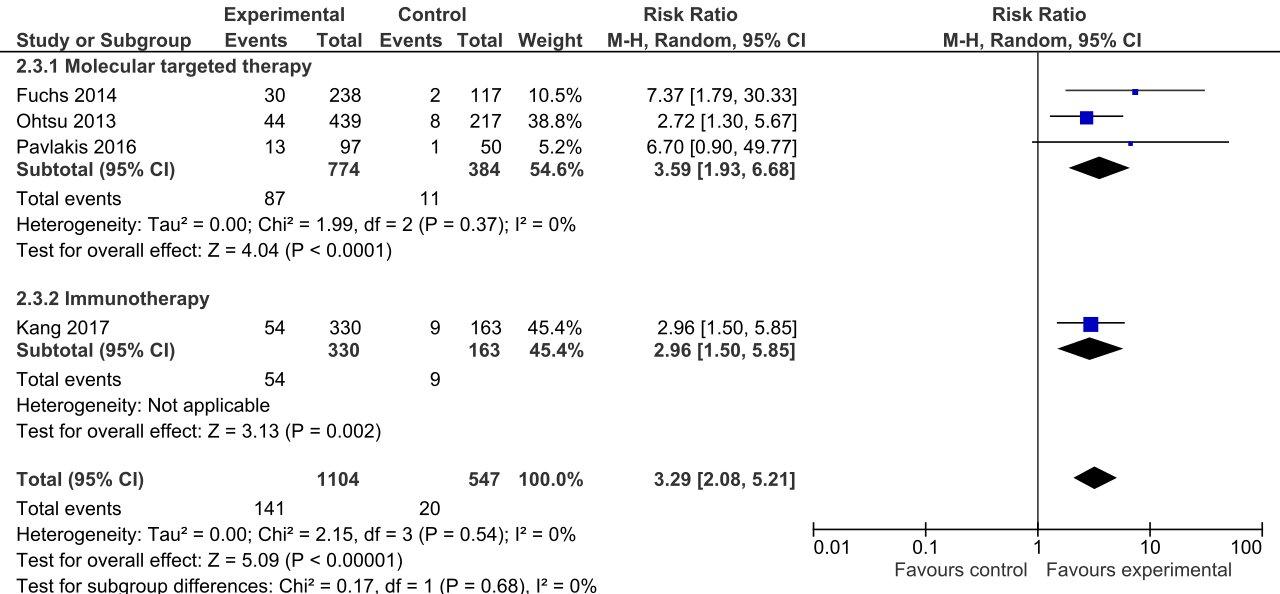


PFS follow-up 12 months


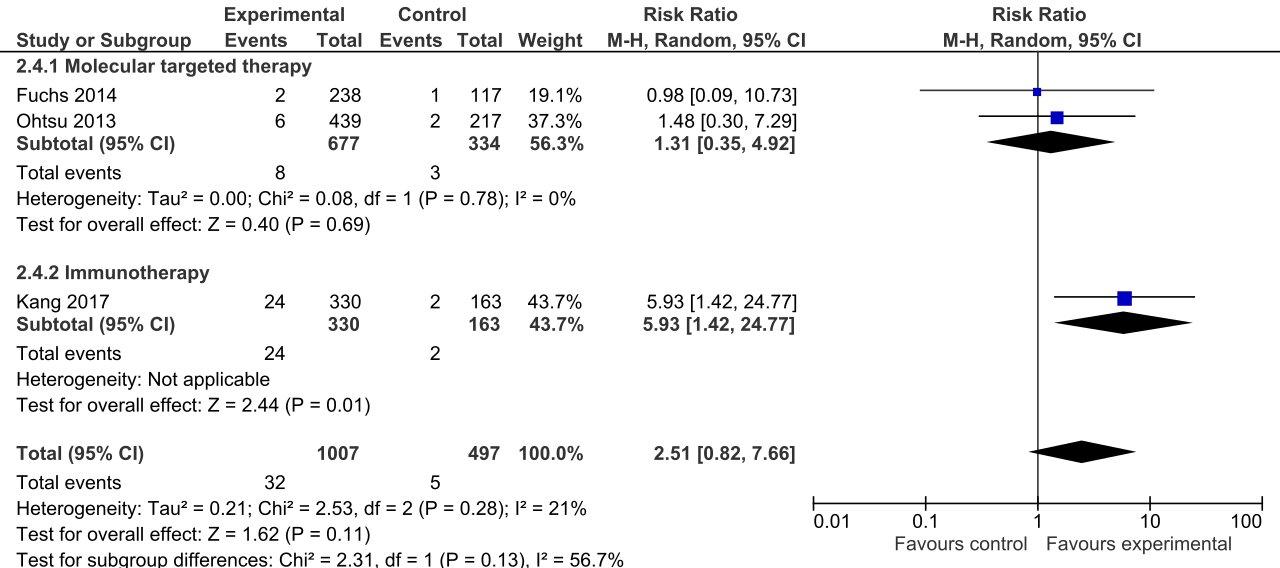


PFS follow-up 18 months


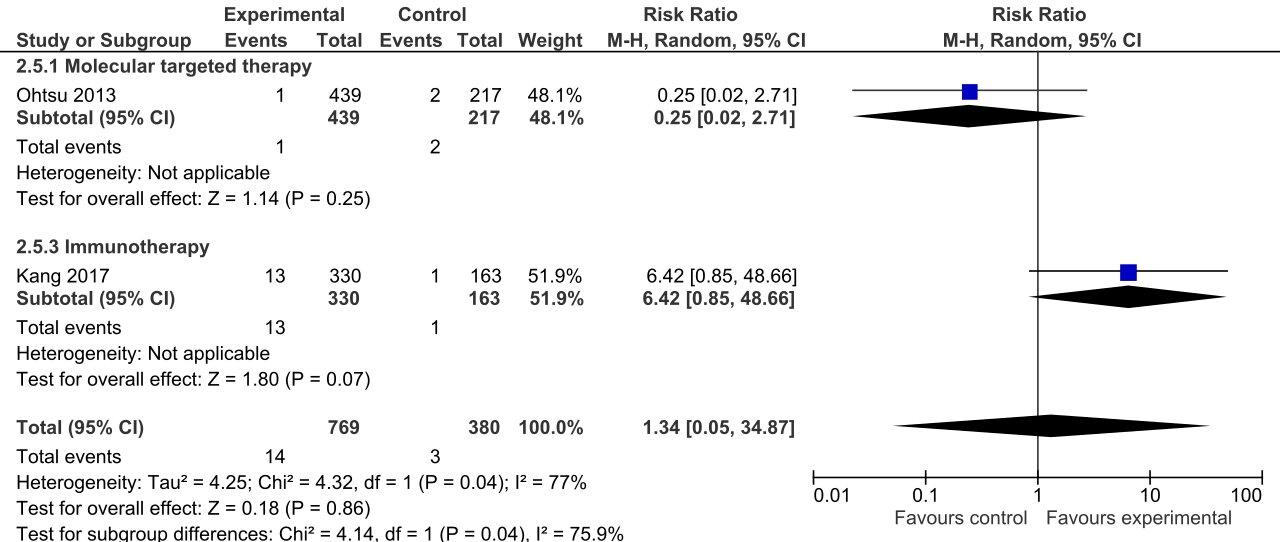


# QoL

# **Appendix 7. Quality of life: results**

| **Study** | **Intervention** | **Scales** | **Follow-up** | **N** | **Description** | **Conclusion** |
| --- | --- | --- | --- | --- | --- | --- |
| Fuchs 2014 | Ramucirumab + BSC | EORTC QLQ-C30 | Baseline | 230/110 | patient-reported global quality of life 6 weeks after the start of treatment initiation | ramucirumab group reported stable or improved global quality of life than those in the placebo group (p=0·23) |
|  |  |  | 6 weeks | 114/29 |  |  |
| Kang 2019 | Rivoceranib + BSC | EORTC QLQ-C30; EORTC QLQ-STO22; EQ-5D-5L | Baseline | 308/152 | change from baseline in Global QoL (EORTC QLQ-C30); change from baseline in EORTC QLQ-STO22 Score; change from baseline in EQ-5D-5L VAS Score; number of participants per QoL dimension response as measured by EQ-5D-5L | NR |
|  |  |  | 24 months | 207/99 |  |  |
| Li 2016 | Apatinib | EORTC QLQ-C30 | Baseline | 176/91 | QoL was assessed at baseline (7 days before first dose of study drug), after cycles two (8 weeks) and three (12 weeks), and every two cycles there after until disease progression, death, or withdrawal of consent,  whichever occurred first | there were no significant differences between the two groups at any time point with regard to QoL score for the different parameters of the EORTC QLQ-C30 (p >0.05) |
|  |  |  | 2 months | 107/43 |  |  |
|  |  |  | 3 months | 61/7 |  |  |
| Ohtsu 2013 | Everolimus +BSC | EORTC QLQ-C30 | Baseline | 439/217 | time to definitive 5% deterioration in the global health status/ QoL and physical, social, and emotional functioning scales | a trend for a slightly longer time to 5% deterioration in global QoL was observed for everolimus (median time to 5% deterioration, 1.51 months v 1.45 months; HR, 0.84; 95% CI, 0.69 to 1.03; P .094).  over time and versus placebo, everolimus recipients  had higher mean scores for the global health status/QoL scale of the QLQ-C30 questionnaire |
|  |  |  | 12 months | NR |  |  |
| Pavlakis 2016 | Regorafenib + BSC | EORTC QLQ-C30; EORTC QLQ-STO2218; EQ-5D | Baseline | 88/48 | QLQ-C30 Global Health Subscale mean estimates | for regorafenib versus placebo were 53 (95% CI, 48 to 58) versus 58 (95% CI, 51 to 65) at week 4 and 54 (95% CI, 48 to 60) versus 56 (95% CI, 45 to 67) at week 8, respectively |
|  |  |  | 1 month | 63/29 |  |  |
|  |  |  | 2 months | 41/11 |  |  |

BSC: best Supportive care; EORTC QLQ-C30: European Organization for Research and Treatment of Cancer Quality of Life Questionnaire-Core 30

# **Appendix 8. GRADE assessment**

**Question: Molecular targeted therapy compared to best supportive care for advanced gastric cancer**

| **Certainty assessment** | | | | | | | **№ of patients** | | **Effect** | | **Certainty** |
| --- | --- | --- | --- | --- | --- | --- | --- | --- | --- | --- | --- |
| **№ of studies** | **Study design** | **Risk of bias** | **Inconsistency** | **Indirectness** | **Imprecision** | **Other considerations** | **Molecular targeted therapy** | **Best supportive care** | **Relative  (95% CI)** | **Absolute  (95% CI)** |  |
| **Overall survival (assessed with: HR)** | | | | | | | | | | | |
| 5 | randomised trials | not serious^a^ | not serious^b^ | not serious^c^ | serious^d^ | none | 1258 participants | 627 participants | **HR 0.84**  (0.75 to 0.93)  [Death] | **64 fewer per 1.000**  (from 26 fewer to 105 fewer) | ⨁⨁⨁◯  Moderate |
|  |  |  |  |  |  |  | - | 30.0%[37] |  | **64 fewer per 1.000**  (from 26 fewer to 105 fewer) |  |
| **Progression free survival (assessed with: HR)** | | | | | | | | | | | |
| 5 | randomised trials | not serious^a^ | serious^e^ | not serious^c^ | not serious | none | 1258 participants | 627 participants | **HR 0.52**  (0.43 to 0.62)  [Death or progression] | **202 fewer per 1.000**  (from 272 fewer to 140 fewer) | ⨁⨁⨁◯  Moderate |
|  |  |  |  |  |  |  | - | 10.0%[38,39] |  | **202 fewer per 1.000**  (from 272 fewer to 140 fewer) |  |
| **Toxicity (assessed with: Adverse events grade 3 or higher)** | | | | | | | | | | | |
| 3 | randomised trials | not serious^a^ | serious^f^ | not serious^c^ | serious^g^ | none | 509/770 (66.1%) | 207/380 (54.5%) | **RR 1.19**  (0.95 to 1.48) | **103 more per 1.000**  (from 27 fewer to 261 more) | ⨁⨁◯◯  Low |
| **Quality of life** | | | | | | | | | | | |
| 4 | randomised trials | serious^h^ | not serious | not serious^c^ | not serious | none | No significant differences between groups. Three studies (Fuchs 2014, Pavlakis 2016 and Kang 2019) showed no significant differences in QoL measured with EORTC QLQ-C30. One study (Ohtsu 2013) showed a non significant longer time to 5% deterioration in global QoL measured with EORTC QLQ-C30 in favour of intervention. | | | | ⨁⨁⨁◯  Moderate |

**CI:** confidence interval; **HR:** hazard ratio; **RR:** risk ratio

**Explanations**

a. One study (Kang 2019) had unclear risk of selection, detection and attrition bias, and other study (Pavlakis 2016) had high risk of attrition bias. Nevertheless, we decided not to downgrade certainty of evidence due to risk of bias, because a sensitivity analysis excluding these studies did not significantly changed the results

b. We did not downgrade any level of certainty of evidence due to inconsistency. There is a low heterogeneity (I2=0%) determined by molecular targeted therapy

c. Although indirectness was considered not serious, all the included studies considered population with ECOG 0-1. Therefore, if the results are considered to be extrapolated to a population with ECOG 2+, the certainty of evidence should be rated down due to indirectness

d. We downgraded one level of certainty of evidence assuming a MID threshold of 10%, the 95%CI around the pooled estimate of the effect crosses the threshold to the left side. MID thresholds: 0.1 corresponds to a small effect; 0.25 corresponds to a moderate effect; 0.4 corresponds to a large effect. Translated into time, the minimum effect is one week; moderate effect is two to three weeks and maximum effect is one month.

e. We downgraded one level of certainty of evidence due to moderate heterogeneity (I2=64%)

f. We downgraded one level of certainty of evidence due to moderate heterogeneity (I2=72%)

g. We downgraded one level of certainty of evidence assuming a MID threshold of 10%, the 95%CI around the pooled estimate of the effect crosses the threshold to the right side

h. We downgraded one level of certainty of evidence due to the unclear risk of performance (Ohtsu 2013) and detection bias (Ohtsu 2013, Kang 2019) and high risk of attrition bias (Pavlakis 2016)

**Question: Immunotherapy compared to best supportive care for advanced gastric cancer**

| **Certainty assessment** | | | | | | | **№ of patients** | | **Effect** | | **Certainty** |
| --- | --- | --- | --- | --- | --- | --- | --- | --- | --- | --- | --- |
| **№ of studies** | **Study design** | **Risk of bias** | **Inconsistency** | **Indirectness** | **Imprecision** | **Other considerations** | **Immunotherapy** | **Best supportive care** | **Relative  (95% CI)** | **Absolute  (95% CI)** |  |
| **Overall survival (assessed with: HR)** | | | | | | | | | | | |
| **1** | **randomised trials** | **not serious** | **not serious** | **not serious^a^** | **serious^b^** | **none** | **330 participants** | **163 participants** | **HR 0.62  (0.51 to 0.75)  [Death]** | **174 fewer per 1.000  (from 241 fewer to 105 fewer)** | ⨁⨁⨁◯  Moderate |
|  |  |  |  |  |  |  | **-** | **30.0%** |  | **174 fewer per 1.000  (from 241 fewer to 105 fewer)** |  |
| Progression free survival | | | | | | | | | | | |
| **1** | **randomised trials** | **not serious** | **not serious** | **not serious^a^** | **serious^b^** | **none** | **330 participants** | **163 participants** | **HR 0.60  (0.49 to 0.73)  [Death or progression]** | **151 fewer per 1.000  (from 86 fewer to 224 fewer)** | ⨁⨁⨁◯  Moderate |
|  |  |  |  |  |  |  | **-** | **10.0%** |  | **151 fewer per 1.000  (from 86 fewer to 224 fewer)** |  |
| Toxicity (assessed with: Adverse event grade 3 or higher) | | | | | | | | | | | |
| **1** | **randomised trials** | **not serious** | **not serious** | **not serious^a^** | **serious^b^** | **none** | **39/330 (11.8%)** | **7/161 (4.3%)** | **RR 2.72  (1.24 to 5.94)** | **75 more per 1.000  (from 10 more to 215 more)** | ⨁⨁⨁◯  Moderate |
| **Quality of life - not reported** | | | | | | | | | | | |
| **-** | **-** | **-** | **-** | **-** | **-** | **-** | **-** | **-** | **-** | **-** | **-** |

**CI: confidence interval; HR: hazard ratio; RR: risk ratio**

Explanations

a. Although indirectness was considered not serious, all the included studies considered population with ECOG 0-1. Therefore, if the results are considered to be extrapolated to a population with ECOG 2+, the certainty of evidence should be rated down due to indirectness

b. We downgraded one level of certainty of evidence due to imprecision due to optimal information size not met.
